# Association Between an Incomplete Circle of Willis and Acute Ischemic Stroke Severity at Admission: A Computed Tomography Angiography Study From Nepal

**DOI:** 10.7759/cureus.116843

**Published:** 2026-09-24

**Authors:** Priyesh Shrestha, Mukunda s Shrestha, Adarsha Mahaseth, Grisha Gurung, Prajwal Dhakal, Ghanshyam Gurung

**Affiliations:** 1 Department of Radiology, Nepal APF (Armed Police Force) Hospital, Kathmandu, NPL; 2 Department of Radiodiagnosis, College of Medicine, Nepalese Army Institute of Health Sciences, Kathmandu, NPL; 3 Department of Internal Medicine, College of Medicine, Nepalese Army Institute of Health Sciences, Kathmandu, NPL; 4 Department of Paediatrics, Baidya and Banskota Hospital, Kathmandu, NPL; 5 Department of Radiology, Tribhuvan University Institute of Medicine, Kathmandu, NPL

**Keywords:** acute ischemic stroke, anatomical variants, circle of willis, collateral circulation, computed tomography angiography, nihss, stroke severity

## Abstract

Background

The Circle of Willis (CoW) is an important collateral pathway during arterial occlusion, but the clinical relevance of common anatomical variants in acute ischemic stroke remains uncertain. This study evaluates whether an incomplete CoW is associated with greater neurological severity at admission and describes CoW morphology on computed tomography angiography (CTA).

Methods

This hospital-based cross-sectional observational analytic study included 190 patients aged >50 years with first-ever acute ischemic stroke presenting within seven days of symptom onset and undergoing head-and-neck CTA at a teaching hospital in Kathmandu, Nepal. CoW morphology was classified using the Hartkamp framework; Type I was considered complete and Types II-IV incomplete. National Institutes of Health Stroke Scale (NIHSS) score at admission was the primary outcome, and NIHSS >15 was a secondary outcome. Multivariable linear and logistic regression and sensitivity analyses were performed.

Results

Among 462 patients assessed, 190 were enrolled and analyzed. Median age was 65 years (interquartile range (IQR) 60-70), and 109 (57.4%) were male. A complete CoW was present in 47 patients (24.7%) and an incomplete CoW in 143 (75.3%); Type II was most frequent (55.3%). Median NIHSS score was higher with incomplete than complete CoW, 10 (IQR 6-16) versus 8 (IQR 6-11), p=0.027. In the adjusted linear model, incomplete CoW was associated with a 1.877-point higher NIHSS score (95% CI 0.164-3.590; p=0.032), although the effect was modest and model explanatory power was limited (adjusted R²=0.073). There was no significant NIHSS gradient across Hartkamp Types I-IV (p=0.167). In the anterior-circulation-only analysis, the adjusted coefficient was 1.810 (95% CI -0.023 to 3.643; p=0.053). Timing-restricted analyses showed similar or slightly larger coefficients. Exploratory regional morphology analysis was not significant overall (p=0.079). For NIHSS score >15, incomplete CoW was associated with higher odds in both the five-predictor model (aOR=3.79, 95% CI 1.23-11.64; p=0.020) and parsimonious model (aOR=3.65, 95% CI 1.20-11.14; p=0.023), although CIs were wide.

Conclusions

Incomplete CoW anatomy was common and was associated with higher NIHSS score at admission after adjustment, but the primary continuous effect was modest. The absence of a monotonic Hartkamp severity gradient, non-significant regional analyses, and attenuation in the anterior-circulation model argue against using binary CoW completeness as a stand-alone marker of collateral capacity. The NIHSS score >15 association was imprecise and should be interpreted cautiously. CoW morphology should be considered within the broader vascular and clinical context.

## Introduction

Stroke remains one of the major causes of death and disability worldwide. Global Burden of Disease 2021 estimates summarized by the World Stroke Organization indicate approximately 11.9 million incident strokes, 93.8 million people living after stroke, and more than 7.2 million stroke-related deaths in 2021; stroke remained the second leading cause of death and the third leading cause of death and disability combined [1]. In Nepal, population-level data remain limited, but a systematic review found that ischemic stroke accounted for approximately 70.9% of reported strokes, while a community study summarized in that review reported crude and age-standardized stroke prevalence of 2,368 and 2,967 per 100,000 population, respectively, in southwestern Nepal [2]. South-East Asia additionally carries a high vascular risk-factor burden and uneven access to organized acute stroke services [3].

The Circle of Willis (CoW) is an arterial anastomotic network connecting the anterior and posterior cerebral circulations. Its communicating segments provide potential alternative routes of blood flow when a major intracranial artery becomes compromised. The hemodynamic contribution of the CoW depends on the caliber and patency of its communicating vessels and on pressure gradients across the network [4]. Anatomical variation is common, particularly in the posterior communicating arteries, and a complete configuration is rare in many angiographic series [5-7].

The clinical effect of these variants during ischemic stroke remains uncertain. Some studies have associated incomplete CoW configurations with greater neurological deficits or worse functional outcome, whereas systematic reviews and treatment-specific cohorts have shown heterogeneous or null associations [8-12]. These differences may reflect variation in study populations, imaging modalities, definitions of completeness, occlusion sites, reperfusion treatment, and whether structural anatomy or functional collateral flow was evaluated.

Nepalese computed tomography angiography (CTA) and magnetic resonance angiography (MRA) studies have documented frequent CoW variants, with posterior communicating artery abnormalities and fetal-origin posterior cerebral artery configurations among the common findings [13-15]. However, local evidence directly relating CoW completeness to neurological severity at admission in acute ischemic stroke is limited.

The primary objective of this study was to compare National Institutes of Health Stroke Scale (NIHSS) score at admission, treated as a continuous outcome, between patients with complete and incomplete CoW configurations and to determine whether CoW incompleteness remained associated with admission NIHSS after adjustment for measured covariates. We hypothesized that an incomplete CoW would be associated with higher NIHSS score at admission, with an anticipated standardized effect of approximately Cohen's d = 0.50 used for sample-size planning. Secondary objectives were to estimate the prevalence and pattern of CoW anatomical variants and to evaluate NIHSS >15. Additional analyses examined whether the primary association was robust in anterior-circulation stroke and in patients assessed earlier after symptom onset, and whether regional anterior versus posterior CoW incompleteness provided additional mechanistic information.

## Materials and methods

This was a hospital-based, cross-sectional, observational analytic study was conducted in the Department of Radiology and the Department of Neuromedicine at Tribhuvan University Teaching Hospital, Institute of Medicine, Kathmandu, Nepal, from October 2, 2024, to September 28, 2025. The study was approved by the Institutional Review Committee of the Institute of Medicine, Tribhuvan University (reference number: 244 081/082, dated October 2, 2024). Written informed consent was obtained from each participant or a legal guardian before enrollment. No additional imaging or invasive procedure was performed solely for research purposes, and patient confidentiality was maintained. The manuscript was prepared in accordance with the Strengthening the Reporting of Observational Studies in Epidemiology (STROBE) recommendations [16]. 

Study population

Eligibility Criteria

Eligible participants were patients aged >50 years with first-ever acute ischemic stroke who presented within seven days of symptom onset or last-known-well, underwent head-and-neck CTA at the study center during the study period, and provided written informed consent directly or through a legal guardian. The seven-day window was used to capture acute stroke presentations reaching the tertiary study center, including delayed and interfacility referrals; because neurological severity can change over this interval, onset-to-assessment timing was characterized and timing-restricted sensitivity analyses were performed.

Patients were excluded if they were aged ≤50 years; presented beyond seven days; did not undergo CTA at the study center or had an inadequate angiographic work-up; had a previous cerebral infarction or intracranial hemorrhage; had received intravenous thrombolysis, mechanical thrombectomy, vascular stenting, or neurosurgical intervention before documentation of the baseline NIHSS score and CTA findings; had another neurological diagnosis; had an intracranial tumor, arteriovenous malformation, cerebral venous sinus thrombosis, or multiple sclerosis; or declined consent. Eligible patients were enrolled consecutively.

Flow of Participants

Of 462 patients assessed for eligibility, 272 were not included, and 190 were enrolled. All 190 enrolled participants were included in the analysis, with no post-enrollment exclusions and no missing values for variables used in the principal regression models. The participant flow and reasons for non-inclusion are shown in Figure 1.

**Figure 1:**
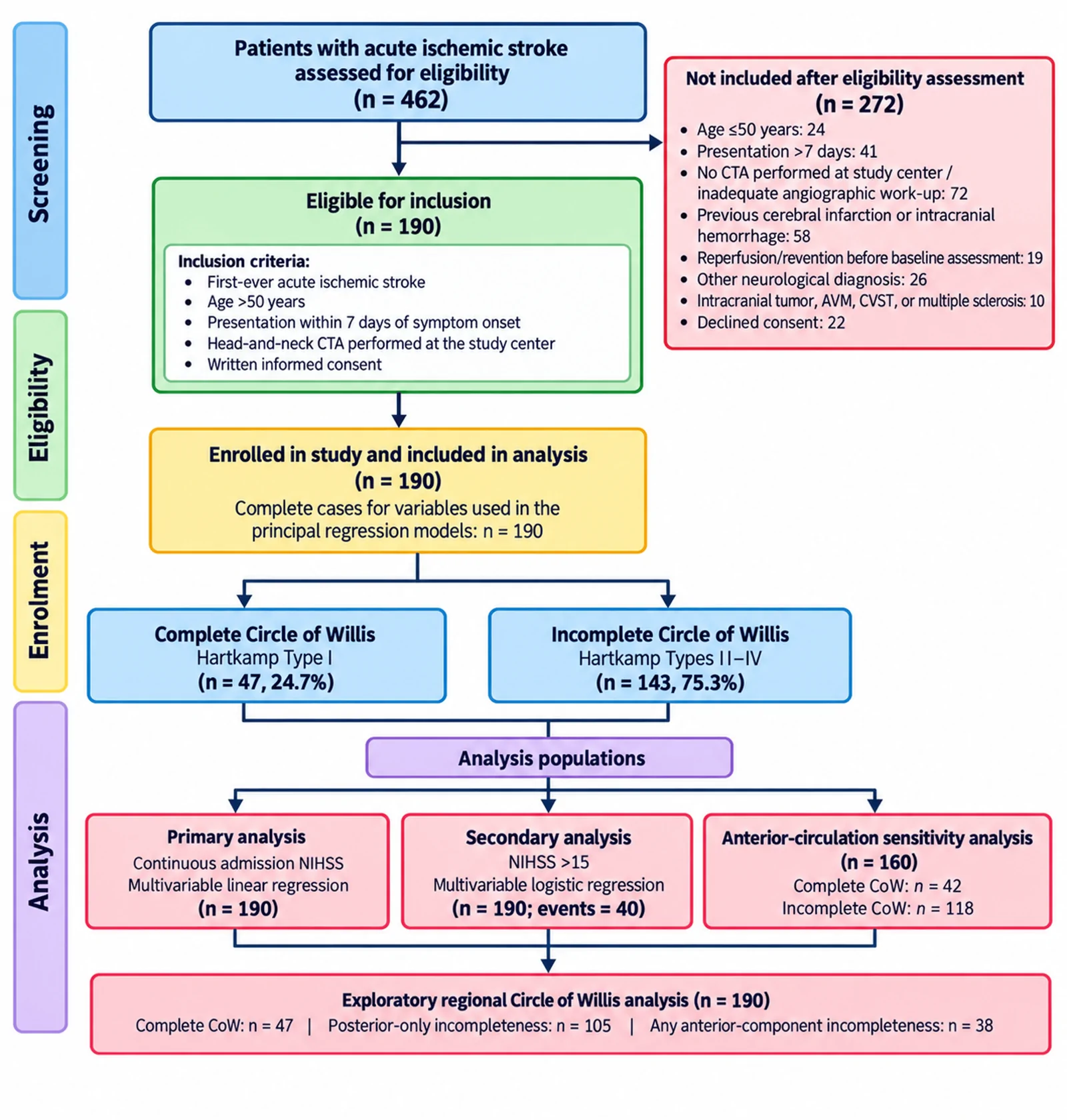
Participant flow diagram A total of 462 patients with acute ischemic stroke were assessed for eligibility; 190 met the study criteria and were enrolled and included in the analysis. The chart also summarizes the recorded reasons for non-inclusion and the populations used in the primary, secondary, anterior-circulation, and exploratory regional analyses. CTA: computed tomography angiography

Sample Size Calculation

The sample size was based on the study by Chowdhury et al., which reported mean NIHSS scores of 6.60 ± 4.97 in patients with a complete CoW and 9.95 ± 5.10 in those with an incomplete CoW [17]. The corresponding observed standardized effect size (Cohen's d) was approximately 0.66. Because the reference study was small, 75% of this effect was used as a conservative planning value, giving an anticipated effect size of approximately 0.50. The planning calculation used a two-sample continuous-outcome comparison with a two-sided alpha of 0.05, 90% power, and equal allocation because reliable local data on the expected complete-to-incomplete CoW ratio were not available at protocol development. This required approximately 170 participants; the target was set at 171 evaluable participants. After allowing approximately 10% for incomplete or non-evaluable data, the planned enrollment was 190. The calculation was therefore powered for the primary continuous NIHSS score comparison, not for the secondary NIHSS score >15 analysis. The observed allocation was more imbalanced than the planning assumption, with 47 complete and 143 incomplete CoW cases.

CTA protocol

CTA was performed using SOMATOM Definition AS or SOMATOM go.Top multidetector CT scanners (Siemens Healthineers AG, Erlangen, Germany), operating on 64-detector/128-slice platforms. Tube voltage ranged from 80 to 100 kVp with automated tube-current modulation (28-665 mA). Non-ionic iodinated contrast medium (70-90 mL) was administered intravenously at a rate of 4-4.5 mL/s. Scan coverage extended from below the aortic arch to the cranial vault. The clinical acquisition protocol used a nominal slice thickness of 0.625 mm with 128-slice acquisition, and image acquisition was performed approximately 8-10 seconds after contrast administration. CTA datasets were subsequently reconstructed and post-processed using multiplanar reformations, maximum-intensity projection (MIP), and volume-rendering techniques (VRT) for vascular assessment.

CoW assessment

CoW anatomy was assessed in the CT reporting room by the performing radiology resident and confirmed by the supervising radiologist. CoW morphology was classified according to the Hartkamp completeness framework, operationalized into four configurations as previously described [6,8]: Type I, complete anterior and posterior components; Type II, complete anterior and incomplete posterior components; Type III, incomplete anterior and complete posterior components; and Type IV, incomplete anterior and posterior components. For the principal analysis, Type I was categorized as complete and Types II-IV as incomplete. Recorded variants included PCom hypoplasia/aplasia, fetal-origin posterior cerebral artery (PCA) with a hypoplastic or absent P1 segment, A1 hypoplasia, anterior communicating artery (ACom) hypoplasia/aplasia, and combined abnormalities.

CoW morphology was evaluated using the thin-section CTA source images in conjunction with MIP and volume-rendered reconstructions. In accordance with the predefined classification criteria, a visible CoW arterial segment with a diameter <0.8 mm was classified as hypoplastic, whereas a segment that was not visualized along its expected anatomical course was classified as aplastic. Source images and vascular reconstructions were reviewed together to assess vessel caliber, continuity, and configuration. Equivocal cases were reviewed jointly by the reporting radiology resident and supervising radiologist before final classification.

Vessel caliber assessment was performed as part of routine CTA interpretation using the thin-section source dataset and vascular reconstructions. Because a separate standardized quantitative caliper-measurement protocol was not prospectively specified or recorded, classification of segments close to the 0.8-mm threshold was based on the integrated morphological assessment of the available CTA images.

Developmental hypoplasia or aplasia was differentiated from acquired stenosis or acute arterial occlusion by reviewing vessel course and morphology on thin-section source images together with multiplanar and maximum-intensity-projection reconstructions. Smooth, uniformly small-caliber vessels or complete non-visualization along the expected course were considered more consistent with developmental variation, whereas abrupt cutoff, focal luminal narrowing, irregularity, atherosclerotic change, or other features suggesting acquired vascular disease were not classified as congenital CoW hypoplasia or aplasia. Uncertain cases were reviewed jointly with the supervising radiologist. The final CoW classification was performed without reference to the numeric NIHSS score at admission. However, the interpreting radiologists were not formally blinded to all other clinical information available during routine acute-care CTA interpretation.

Stroke severity, timing, and clinical variables

Stroke severity was assessed at hospital admission using the NIHSS by a neurology resident or treating physician trained in NIHSS assessment, before thrombolytic therapy or another acute reperfusion intervention whenever applicable. Scores were categorized as minor (0-4), moderate (5-15), moderate-to-severe (16-20), and severe (21-42). NIHSS score at admission as a continuous variable was the prespecified primary outcome. For the secondary binary analysis, NIHSS score ≤15 was classified as non-severe/mild-to-moderate and NIHSS score >15 as greater neurological severity.

Demographic characteristics, smoking history, hypertension, diabetes mellitus, atrial fibrillation/flutter, valvular heart disease, rheumatic heart disease, stroke laterality, and vascular territory were recorded using a structured proforma. For the revision analyses, the available clinical records were also used to characterize symptom-onset or last-known-well time, hospital arrival time, NIHSS assessment time, CTA time, interfacility referral status, and whether timing was based on last-known-well or uncertain/wake-up onset.

Statistical analysis

Data were analyzed using IBM SPSS Statistics for Windows, version 29 (IBM Corp., Armonk, New York, United States). Continuous variables were examined for normality using the Shapiro-Wilk test. Because overall NIHSS scores were non-normally distributed, medians and quartiles were emphasized, and non-parametric tests were used for principal unadjusted comparisons. The Mann-Whitney U test was used for two-group comparisons and the Kruskal-Wallis test for variables with more than two categories. Categorical variables were compared using Pearson chi-square or Fisher's exact test as appropriate. Exploratory Spearman analyses were used as rank-based association measures for age and binary predictors; because several predictors were binary, these correlations were interpreted descriptively rather than as conventional ordinal-ordinal correlations. Hartkamp Types I-IV were treated as nominal morphological categories rather than an ordinal severity scale.

Multivariable linear regression evaluated NIHSS score at admission after adjustment for age, sex, smoking history, hypertension, diabetes mellitus, atrial fibrillation/flutter, and CoW integrity. A sensitivity model additionally included valvular and rheumatic heart disease. Regression assumptions were examined using the residual histogram, normal P-P plot, standardized residual-versus-predicted plot, and case-wise residuals. Multicollinearity was assessed using tolerance and variance inflation factor statistics. Influence was evaluated using Cook's distance, centered leverage, and studentized residuals. Robustness of the primary linear model was also assessed with 5,000 bootstrap samples.

Prespecified/reviewer-requested sensitivity analyses repeated the primary adjusted model among anterior-circulation strokes and among participants assessed within 48 hours and within 24 hours of symptom onset or last-known-well. To examine anatomical location, participants were also grouped as complete CoW, posterior-only incompleteness, or any anterior-component incompleteness; overall Kruskal-Wallis testing and adjusted incremental model testing were used before interpreting individual contrasts.

Binary logistic regression evaluated NIHSS score >15 using age, hypertension, diabetes mellitus, atrial fibrillation/flutter, and CoW integrity. Because only 40 events were observed, corresponding to approximately eight events per predictor in the five-predictor model, a parsimonious sensitivity model containing only CoW integrity and atrial fibrillation/flutter was also examined. Effect estimates are reported with 95% confidence intervals (CIs). Complete-case analysis was used for the principal models. A two-sided p-value <0.05 was used for the prespecified primary analysis. No formal multiplicity adjustment was applied to secondary or exploratory analyses; these results were interpreted descriptively with emphasis on effect estimates, CIs, and consistency rather than isolated p-values.

## Results

Demographic and clinical characteristics

A total of 190 patients with first-ever acute ischemic stroke were included in the analysis, and all had complete data for variables used in the principal linear and logistic regression models. The median age was 65 years (Q1-Q3: 60-70 years), with a range of 52-85 years. There were 109 male patients (57.4%) and 81 female patients (42.6%). Hypertension was the most frequent vascular risk factor (n=112, 58.9%), followed by smoking history (n=190, 28.9%), diabetes mellitus (n=45, 23.7%), and atrial fibrillation/flutter (n=20, 10.5%). The median NIHSS score at admission was 9 (Q1-Q3: 6-13), and 40 patients (21.1%) had an NIHSS score >15. Anterior-circulation stroke predominated (n=160, 84.2%). Detailed demographic, vascular-risk, stroke-territory, and severity characteristics are summarized in Table 1.

**Table 1:** Demographic, clinical, and stroke characteristics of the study population (N=190) NIHSS: National Institutes of Health Stroke Scale; Q1: first quartile; Q3: third quartile

Characteristics		Value
Age (years)	Median (Q1-Q3)	65 (60-70)
	Range	52-85
	51-60, n (%)	55 (28.9%)
	61-70, n (%)	89 (46.8%)
	71-80, n (%)	42 (22.1%)
	81-85, n (%)	4 (2.1%)
Sex, n (%)	Male	109 (57.4%)
	Female	81 (42.6%)
Smoking history, n (%)		55 (28.9%)
Comorbities, n (%)	Hypertension	112 (58.9%)
	Diabetes mellitus	45 (23.7%)
	Atrial fibrillation/flutter	20 (10.5%)
	Valvular heart disease	14 (7.4%)
	Rheumatic heart disease	8 (4.2%)
NIHSS Score	Median (Q1-Q3)	9 (6-13)
	Range	0-25
	0-4 (Minor stroke), n (%)	30 (15.8%)
	5-15 (Moderate stroke), n (%)	120 (63.2%)
	16-20 (Moderate-to-severe stroke), n (%)	34 (17.9%)
	21-42 (Severe stroke), n (%)	6 (3.2%)
	≤15, n (%)	150 (78.9%)
	>15, n (%)	40 (21.1%)
Stroke classification, n (%)	Right-sided stroke	90 (47.4%)
	Left-sided stroke	94 (49.5%)
	Bilateral stroke	2 (1.1%)
	Multifocal stroke	4 (2.1%)
	Anterior-circulation stroke	160 (84.2%)
	Posterior-circulation stroke	23 (12.1%)
	Both circulations	7 (3.7%)

CoW morphology and anatomical variants

A complete Hartkamp Type I CoW was identified in 47 patients (24.7%), whereas 143 (75.3%) had an incomplete CoW. Type II morphology, characterized by a complete anterior and incomplete posterior component, was the most frequent configuration (105/190, 55.3%). Type III occurred in 10 patients (5.3%) and Type IV in 28 (14.7%). Bilateral PCom hypoplasia/aplasia was the most frequent individual recorded abnormality (31/190, 16.3%). The Hartkamp distribution and the mutually exclusive variant catalogue are shown in Table 2. Representative CTA examples of the four Hartkamp configurations are shown in Figure 2.

**Table 2:** Circle of Willis morphology and mutually exclusive recorded anatomical variants Specific variant categories are mutually exclusive; combined abnormalities were coded separately rather than counted repeatedly under their component variants. ACom: anterior communicating artery; CoW: Circle of Willis; PCA: posterior cerebral artery; PCom: posterior communicating artery.

Finding		Frequency (Percentage)
Hartkamp morphology and overall completeness	Hartkamp Type I - complete anterior and posterior components	47 (24.7%)
	Hartkamp Type II - complete anterior/incomplete posterior	105 (55.3%)
	Hartkamp Type III - incomplete anterior/complete posterior	10 (5.3%)
	Hartkamp Type IV - incomplete anterior and posterior	28 (14.7%)
	Overall complete CoW	47 (24.7%)
	Overall incomplete CoW	143 (75.3%)
Mutually exclusive specific anatomical variant categories	No recorded variant	47 (24.7%)
	Right PCom hypoplasia/aplasia	13 (6.8%)
	Left PCom hypoplasia/aplasia	14 (7.4%)
	Bilateral PCom hypoplasia/aplasia	31 (16.3%)
	Fetal-origin right PCA with hypoplastic/absent right P1	9 (4.7%)
	Fetal-origin left PCA with hypoplastic/absent left P1	7 (3.7%)
	Bilateral fetal-origin PCA with bilateral hypoplastic/absent P1	10 (5.3%)
	Right PCom hypoplasia + fetal-origin left PCA	11 (5.8%)
	Left PCom hypoplasia + fetal-origin right PCA	10 (5.3%)
	Right A1 hypoplasia	4 (2.1%)
	Left A1 hypoplasia	3 (1.6%)
	ACom hypoplasia/aplasia	3 (1.6%)
	Right A1 hypoplasia + left PCom hypoplasia	6 (3.2%)
	Left A1 hypoplasia + right PCom hypoplasia	6 (3.2%)
	ACom hypoplasia/aplasia + bilateral PCom hypoplasia	5 (2.6%)
	Right A1 hypoplasia + fetal-origin left PCA	4 (2.1%)
	Left A1 hypoplasia + fetal-origin right PCA	3 (1.6%)
	Multiple anterior and posterior anomalies	4 (2.1%)

**Figure 2:**
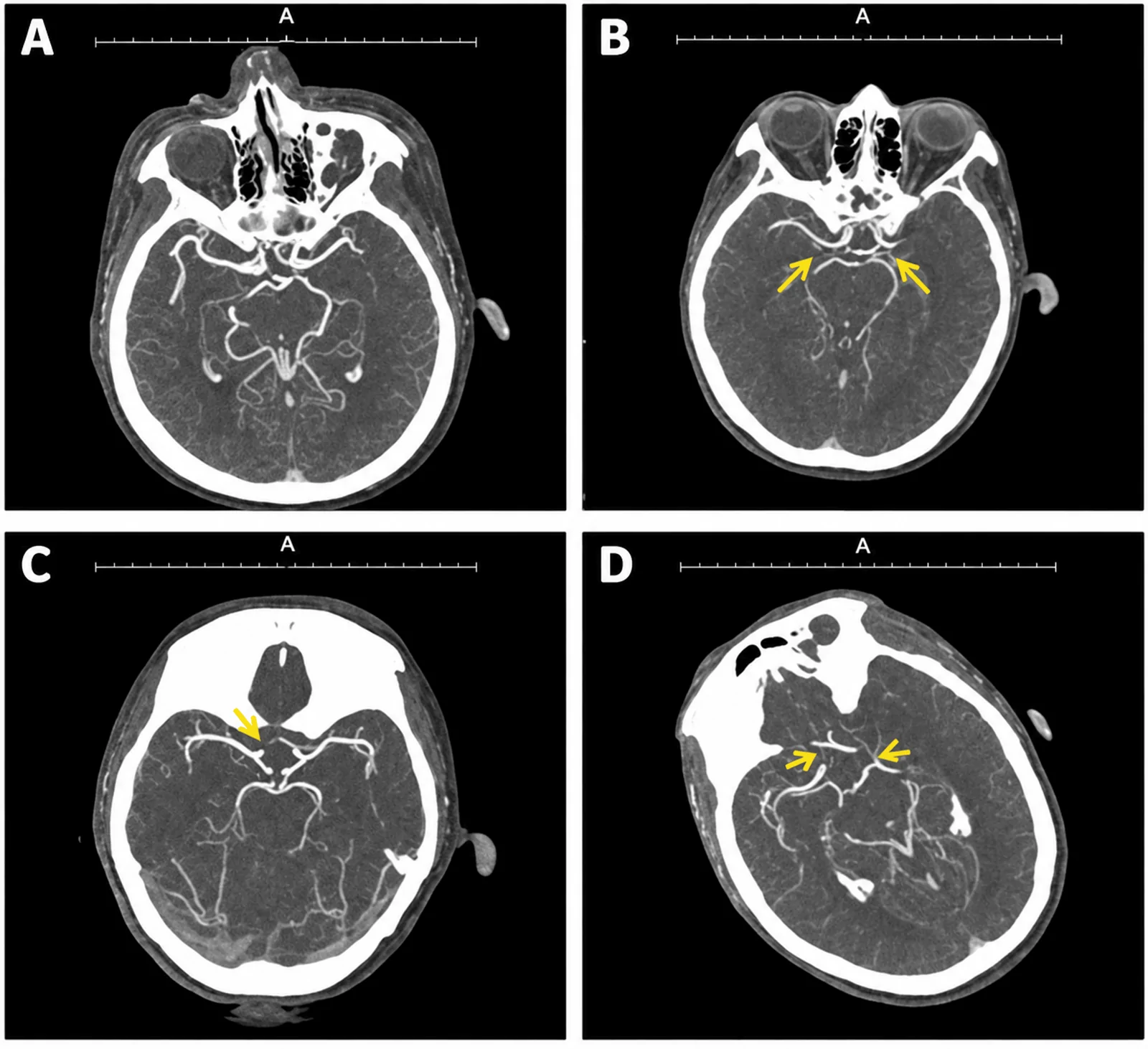
Representative CT angiography images illustrating Hartkamp Circle of Willis configurations (A) Type I complete Circle of Willis in a 71-year-old male patient with acute right-sided ischemic stroke (NIHSS score = 8); (B) Type II configuration in a 63-year-old female patient (NIHSS score = 7), with fetal origin of the left PCA and hypoplastic right PCom; (C) Type III configuration in a 60-year-old female patient with multifocal embolic stroke involving both anterior and posterior circulations (NIHSS score = 10), with a hypoplastic A1 segment of the right ACA; and (D) Type IV configuration in a 66-year-old female patient with acute left-sided ischemic stroke (NIHSS score = 17), with a hypoplastic left PCom and hypoplastic A1 segment of the right ACA. Yellow arrows indicate the relevant Circle of Willis anatomy or abnormality in panels B-D. PCA: posterior cerebral artery; PCom: posterior communicating artery; ACA: anterior cerebral artery; NIHSS: National Institutes of Health Stroke Scale

NIHSS score at admission according to CoW completeness

Overall NIHSS scores were non-normally distributed (Shapiro-Wilk p=0.003); therefore, non-parametric testing was used for the primary unadjusted comparison. Patients with an incomplete CoW had a higher median NIHSS score at admission than those with a complete CoW: 10 (Q1-Q3: 6-16) versus 8 (Q1-Q3: 6-11), respectively (Mann-Whitney U=2636.5, Z=-2.218, p=0.027). The distributions nevertheless overlapped substantially: both groups had a first quartile of 6, while the upper quartile was higher in the incomplete-CoW group (16 versus 11), indicating greater separation toward the upper portion of the distribution. Atrial fibrillation/flutter was also associated with greater severity at admission, while no significant unadjusted NIHSS score differences were observed according to sex, smoking history, hypertension, diabetes mellitus, valvular heart disease, or rheumatic heart disease (Table 3).

**Table 3:** Comparison of mean NIHSS score at admission according to demographic and clinical characteristics All comparisons used the Mann-Whitney U test, *Statistically significant at p<0.05. NIHSS: National Institutes of Health Stroke Scale; Q1: first quartile; Q3: third quartile.

Characteristic	Category	n	Median NIHSS Score (Q1–Q3)	Mann–Whitney U	p-value
Sex	Male	109	9 (6–14)	4372.0	0.910
	Female	81	9 (6–13)		
Smoking history	No	135	9 (6–13)	3346.5	0.286
	Yes	55	10 (6–16)		
Hypertension	No	78	8.5 (5–13)	4161.0	0.578
	Yes	112	9.5 (6–13)		
Diabetes mellitus	No	145	9 (6–13)	3075.0	0.560
	Yes	45	10 (5.5–16)		
Atrial fibrillation/flutter	No	170	9 (5–13)	900.0	<0.001*
	Yes	20	15 (9.25–17)		
Valvular heart disease	No	176	9 (6–13)	1174.5	0.771
	Yes	14	9.5 (6.5–16)		
Rheumatic heart disease	No	182	9 (6–13)	606.5	0.424
	Yes	8	8.5 (2–14.5)		
Circle of Willis integrity	Complete	47	8 (6–11)	2636.5	0.027*
	Incomplete	143	10 (6–16)		

NIHSS score according to Hartkamp morphology and stroke characteristics

NIHSS score at admission did not differ significantly across Hartkamp Types I-IV (Kruskal-Wallis H=5.064, df=3, p=0.167). Median NIHSS score was 8 (Q1-Q3: 6-11) for Type I, 10 (Q1-Q3: 6-16) for Type II, 10.5 (Q1-Q3: 4.75-13.25) for Type III, and 10 (Q1-Q3: 6.25-13) for Type IV. There was therefore no monotonic increase in neurological severity from Type I through Type IV. NIHSS scores also did not differ significantly according to age group (p=0.526), vascular territory (p=0.640), stroke laterality (p=0.184), or the detailed anatomical-variant category (p=0.521).

CoW completeness and categorical stroke severity

When NIHSS was evaluated using the four conventional severity categories, moderate-to-severe stroke occurred in 31 of 143 patients (21.7%) with incomplete CoW compared with three of 47 patients (6.4%) with complete CoW; severe stroke occurred in five (3.5%) and one (2.1%) in patients with incomplete and complete CoW, respectively. The overall four-category association did not reach statistical significance (χ²=7.592, df=3, p=0.055), and this analysis was considered exploratory because of small expected cell counts. For the secondary binary endpoint, 36 of 143 patients (25.2%) with incomplete CoW had NIHSS score >15 compared with four of 47 patients (8.5%) with complete CoW (χ²=5.910, df=1, p=0.015), corresponding to a crude OR of 3.62 (95% CI 1.21-10.78). These distributions are shown in Table 4. The Hartkamp-specific NIHSS score >15 comparison was treated as exploratory because of small subgroup counts and multiple comparisons.

**Table 4:** Circle of Willis completeness and admission NIHSS severity For NIHSS >15 versus ≤15: χ²=5.910, df=1, p=0.015; crude OR=3.62 (95% CI 1.21-10.78). CI: confidence interval; CoW: Circle of Willis; NIHSS: National Institutes of Health Stroke Scale; OR: odds ratio

NIHSS Category	Complete CoW, n (%)	Incomplete CoW, n (%)	Total
Minor, 0-4	6 (12.8%)	24 (16.8%)	30
Moderate, 5-15	37 (78.7%)	83 (58.0%)	120
Moderate-to-severe, 16-20	3 (6.4%)	31 (21.7%)	34
Severe, 21-42	1 (2.1%)	5 (3.5%)	6
NIHSS score ≤15	43 (91.5%)	107 (74.8%)	150
NIHSS score >15	4 (8.5%)	36 (25.2%)	40

Baseline characteristics according to CoW completeness

The complete CoW and incomplete CoW groups were similar with respect to measured baseline demographic and vascular risk characteristics. Median age was 66 years (Q1-Q3: 60-70) in the complete CoW group and 64 years (60-70) in the incomplete CoW group (p=0.588). No significant between-group differences were observed in sex, smoking history, hypertension, diabetes mellitus, atrial fibrillation/flutter, valvular heart disease, rheumatic heart disease, vascular territory, or stroke laterality. The principal baseline comparisons are shown in Table 5.

**Table 5:** Baseline characteristics according to Circle of Willis completeness Age was compared using the Mann-Whitney U test. Pearson chi-square or Fisher exact test was used for categorical variables as appropriate. CoW: Circle of Willis; Q1: first quartile; Q3: third quartile

Characteristics	Complete CoW (n=47)	Incomplete CoW (n=143)	p-value
Age (years), median (Q1-Q3)	66 (60-70)	64 (60-70)	0.588
Male, n (%)	26 (55.3%)	83 (58.0%)	0.743
Smoking history, n (%)	14 (29.8%)	41 (28.7%)	0.884
Hypertension, n (%)	28 (59.6%)	84 (58.7%)	0.920
Diabetes mellitus, n (%)	12 (25.5%)	33 (23.1%)	0.731
Atrial fibrillation/flutter, n (%)	4 (8.5%)	16 (11.2%)	0.786
Valvular heart disease, n (%)	3 (6.4%)	11 (7.7%)	1.000
Rheumatic heart disease, n (%)	2 (4.3%)	6 (4.2%)	1.000

Exploratory rank-based associations with NIHSS score at admission

Exploratory rank-based analyses showed weak positive associations between NIHSS score at admission and atrial fibrillation/flutter (r_s=0.251, p<0.001) and between NIHSS score and incomplete CoW (r_s=0.161, p=0.026). No significant rank-based associations were observed with age, sex, smoking history, hypertension, diabetes mellitus, valvular heart disease, or rheumatic heart disease. Because several predictors were binary and these analyses were exploratory, the multivariable regression models were considered more informative for adjusted inference (Table 6).

**Table 6:** Spearman rank-based associations with admission NIHSS Exploratory analysis. *Statistically significant at p<0.05. No formal multiplicity adjustment was applied. NIHSS: National Institutes of Health Stroke Scale

Variable	Spearman r_s	p-value
Age	0.028	0.704
Female sex	-0.008	0.910
Smoking history	0.078	0.287
Hypertension	0.040	0.579
Diabetes mellitus	0.042	0.561
Atrial fibrillation/flutter	0.251	<0.001*
Valvular heart disease	0.021	0.772
Rheumatic heart disease	-0.058	0.425
Incomplete Circle of Willis	0.161	0.026*

Multivariable analysis of NIHSS score at admission

In univariate linear regression, incomplete CoW was associated with an increase of 1.947 NIHSS points (95% CI 0.189-3.704; p=0.030), and atrial fibrillation/flutter with an increase of 4.624 points (95% CI 2.211-7.036; p<0.001). In the primary multivariable model adjusting for age, sex, smoking history, hypertension, diabetes mellitus, atrial fibrillation/flutter, and CoW integrity, incomplete CoW remained associated with higher NIHSS score at admission (B=1.877, SE=0.868, standardized β=0.152, 95% CI 0.164-3.590; p=0.032). Atrial fibrillation/flutter showed a larger adjusted association (B=4.423, 95% CI 2.003-6.844; p<0.001). The overall model was significant (F(7,182)=3.116, p=0.004), although explanatory capacity was limited (R²=0.107; adjusted R²=0.073). Full coefficients are provided in Table 7.

**Table 7:** Multivariable linear regression for admission NIHSS Model R²=0.107; adjusted R²=0.073; F(7,182)=3.116; p=0.004. B: unstandardized regression coefficient; *Statistically significant at p<0.05. CI: confidence interval; NIHSS: National Institutes of Health Stroke Scale; SE: standard error

Predictor	B	SE	Standardized β	95% CI for B	p-value
Age, per year	0.027	0.053	0.036	-0.078 to 0.132	0.612
Female sex	0.220	0.786	0.020	-1.331 to 1.771	0.780
Smoking history	1.268	0.852	0.108	-0.413 to 2.949	0.138
Hypertension	0.140	0.774	0.013	-1.388 to 1.668	0.857
Diabetes mellitus	0.609	0.884	0.048	-1.136 to 2.353	0.492
Atrial fibrillation/flutter	4.423	1.227	0.254	2.003 to 6.844	<0.001*
Incomplete Circle of Willis	1.877	0.868	0.152	0.164 to 3.590	0.032*

A sensitivity model additionally including valvular heart disease and rheumatic heart disease produced similar estimates. Incomplete CoW remained associated with NIHSS score (B=1.872, 95% CI 0.155-3.588; p=0.033), as did atrial fibrillation/flutter (B=4.619, 95% CI 2.040-7.199; p=0.001). This extended model had R²=0.114 and adjusted R²=0.069. A 5,000-sample bootstrap analysis likewise yielded a similar estimate for incomplete CoW (B=1.877, bootstrap SE=0.786, 95% bootstrap CI 0.298-3.402; p=0.021).

Regression diagnostics and influence analysis

There was no evidence of problematic multicollinearity; the maximum variance inflation factor was 1.081, and the minimum tolerance was 0.925. Residual plots did not demonstrate a major violation of linear-regression assumptions. Additional influence analysis showed a maximum Cook's distance of 0.046, maximum centered leverage of 0.096, and maximum absolute studentized residual of 2.89. No observation had an absolute studentized residual ≥3. Although several observations exceeded conservative screening thresholds for Cook's distance or leverage, none demonstrated extreme influence sufficient to suggest that the primary CoW coefficient was driven by a single observation.

Anterior-circulation sensitivity analysis

Because NIHSS score may underrepresent some posterior-circulation deficits, the primary analysis was repeated among the 160 patients with anterior-circulation stroke. Of these, 42 had a complete CoW, and 118 had an incomplete CoW. In the unadjusted analysis, NIHSS remained higher in the incomplete CoW group (Mann-Whitney U=1949.0, Z=-2.056, p=0.040). After adjustment for the same seven covariates used in the primary model, the magnitude of the incomplete CoW coefficient remained similar to that in the full cohort, although the CI narrowly crossed zero (B=1.810, 95% CI -0.023 to 3.643; p=0.053). The restricted model had R²=0.112 and adjusted R²=0.071 (F(7,152)=2.745; p=0.010).

Regional CoW analysis

To examine whether the anatomical location of incompleteness provided information beyond the binary complete/incomplete classification, patients were categorized into three groups: complete CoW (n=47, 24.7%), posterior-only incompleteness (n=105, 55.3%), and any anterior-component incompleteness (n=38, 20.0%). The overall NIHSS distribution did not differ significantly among these groups (Kruskal-Wallis H=5.064, df=2, p=0.079). In the adjusted linear model, adding posterior-only and anterior-component incompleteness increased R² by 0.025, but the regional morphology terms were not jointly significant (F-change=2.488, p=0.086). Relative to complete CoW, posterior-only incompleteness had an adjusted coefficient of 2.026 NIHSS points (95% CI 0.232-3.820; p=0.027), whereas any anterior-component incompleteness had a coefficient of 1.467 points (95% CI -0.765 to 3.699; p=0.196). Because the overall regional test and incremental model test were not significant, individual contrasts were interpreted descriptively rather than as independent confirmatory findings.

Among patients with anterior-circulation stroke, 42 had complete CoW, 85 had posterior-only incompleteness, and 33 had an anterior-component abnormality. The overall three-group comparison remained non-significant (H=4.504, df=2, p=0.105), and addition of the regional terms did not significantly improve the adjusted model (F-change=2.224, p=0.112). The adjusted coefficients were 2.049 points for posterior-only incompleteness (95% CI 0.123-3.975; p=0.037) and 1.185 points for anterior-component incompleteness (95% CI -1.204 to 3.575; p=0.329), again without evidence of a consistent anatomical severity gradient.

Onset-to-assessment characteristics and timing-restricted sensitivity analyses

NIHSS assessment occurred within 24 hours of symptom onset or last-known-well in 107 patients (56.3%), at 25-48 hours in 38 (20.0%), at 49-72 hours in 23 (12.1%), at 73-120 hours in 18 (9.5%), and at 121-168 hours in four (2.1%). Overall, 145 patients (76.3%) were assessed within 48 hours and 168 (88.4%) within 72 hours. Sixty-three patients (33.2%) were interfacility referrals, and timing was based on last-known-well or uncertain/wake-up onset in 36 (18.9%). The mean onset/last-known-well-to-arrival interval was 31.7 hours, and the mean onset/last-known-well-to-NIHSS interval was 32.1 hours (range 2.2-155.6 hours). NIHSS was assessed a mean of 25.6 minutes after hospital arrival, and CTA a mean of 91.2 minutes after arrival.

Patients with NIHSS score >15 tended to be assessed earlier: 27 of 40 (67.5%) were assessed within 24 hours compared with 80 of 150 (53.3%) with NIHSS score ≤15; however, the overall difference in onset-to-NIHSS timing by binary severity category was not statistically significant (Kruskal-Wallis H≈2.24, p=0.134). In the ≤48-hour analysis (n=145), incomplete CoW was associated with a 2.193-point higher adjusted NIHSS score (95% CI 0.219-4.167; p=0.030); in the ≤24-hour analysis (n=107), the corresponding estimate was 2.612 points (95% CI 0.132-5.093; p=0.039). Atrial fibrillation/flutter remained associated with greater NIHSS score in both restricted models.

Predictors of NIHSS score >15

On univariate logistic regression, atrial fibrillation/flutter was associated with NIHSS score >15 (OR=4.67, 95% CI 1.78-12.20; p=0.002), as was incomplete CoW (OR=3.62, 95% CI 1.21-10.78; p=0.021). In the five-predictor multivariable logistic model, atrial fibrillation/flutter remained associated with NIHSS score >15 (aOR=4.37, 95% CI 1.59-12.02; p=0.004). Incomplete CoW was also associated with the secondary binary outcome (aOR=3.79, 95% CI 1.23-11.64; p=0.020). Age, hypertension, and diabetes mellitus were not independently associated with NIHSS score >15. The model was significant (χ²=20.488, df=5, p=0.001), with Cox-Snell R²=0.102 and Nagelkerke R²=0.159; the Hosmer-Lemeshow test was non-significant (χ²=3.599, p=0.891). Coefficients are shown in Table 8.

**Table 8:** Multivariable logistic regression for NIHSS >15 Model χ²=20.488, df=5, p=0.001; Cox-Snell R²=0.102; Nagelkerke R²=0.159; Hosmer-Lemeshow χ²=3.599, p=0.891. *Statistically significant at p<0.05. CI: confidence interval; NIHSS: National Institutes of Health Stroke Scale; OR: odds ratio; SE: standard error

Predictor	B	SE	Adjusted OR	95% CI	p-value
Age, per year	0.042	0.027	1.04	0.99-1.10	0.119
Hypertension	-0.239	0.392	0.79	0.37-1.70	0.541
Diabetes mellitus	0.623	0.419	1.86	0.82-4.24	0.137
Atrial fibrillation/flutter	1.474	0.516	4.37	1.59-12.02	0.004*
Incomplete Circle of Willis	1.331	0.573	3.79	1.23-11.64	0.020*

Given the limited number of NIHSS score >15 events (40/190), a parsimonious model containing only CoW integrity and atrial fibrillation/flutter was also examined. The estimate for incomplete CoW remained similar (aOR=3.65, 95% CI 1.20-11.14; p=0.023), while atrial fibrillation/flutter had an aOR of 4.71 (95% CI 1.75-12.67; p=0.002). The two-predictor model was significant (χ²(2)=15.96, p<0.001), with Cox-Snell R²=0.081 and Nagelkerke R²=0.125. The persistence of a similar point estimate in the reduced model suggested numerical stability of the association, although the CI remained wide.

Summary of sensitivity and exploratory analyses

Sensitivity analyses are summarized in Table 9. Across these analyses, the complete-versus-incomplete CoW coefficient remained modest in magnitude. It was similar but borderline in the anterior-circulation-only model, while timing-restricted estimates were somewhat larger. Neither Hartkamp subtyping nor regional morphology demonstrated a monotonic or consistently stronger anatomical severity gradient, and regional morphology did not significantly improve the adjusted models. The secondary NIHSS score >15 estimate remained similar in the parsimonious model but was imprecise.

**Table 9:** Summary of sensitivity analyses for Circle of Willis incompleteness Regional individual contrasts were exploratory because the overall regional block was not statistically significant. No formal adjustment for multiple comparisons was applied to secondary or exploratory analyses. B: adjusted linear-regression coefficient in NIHSS points; aOR: adjusted odds ratio; CI: confidence interval.

Analysis	n	Effect estimate	95% CI	p-value	Model note
Primary adjusted linear model	190	B=1.877	0.164 to 3.590	0.032	Adjusted R²=0.073
Anterior-circulation-only model	160	B=1.810	-0.023 to 3.643	0.053	Adjusted R²=0.071
≤48-hour restricted model	145	B=2.193	0.219 to 4.167	0.030	Adjusted R²=0.075
≤24-hour restricted model	107	B=2.612	0.132 to 5.093	0.039	Adjusted R²=0.085
Regional: posterior-only vs complete	190	B=2.026	0.232 to 3.820	0.027	Overall regional block p=0.086
Regional: anterior-component vs complete	190	B=1.467	-0.765 to 3.699	0.196	Overall regional block p=0.086
Parsimonious logistic model	190	aOR=3.65	1.20 to 11.14	0.023	Nagelkerke R²=0.125

## Discussion

Principal findings

The prespecified primary result was a modest association between incomplete CoW and higher NIHSS score at admission. After adjustment for measured covariates, incomplete CoW was associated with a 1.877-point higher NIHSS score (95% CI 0.164-3.590), while the overall model explained only 7.3% of NIHSS variance after adjustment. The lower confidence bound was close to zero, indicating that the data remain compatible with a very small clinical difference. The secondary NIHSS score >15 analysis produced a larger relative effect estimate (aOR=3.79), but this estimate was based on only 40 events and had a wide CI. The primary continuous outcome should therefore carry greater interpretive weight.

Several findings qualify the binary complete-versus-incomplete association. There was no monotonic NIHSS gradient across Hartkamp Types I-IV, the three-group regional analysis was not significant overall, and adding regional morphology did not significantly improve the adjusted model. The anterior-circulation-only coefficient remained similar in magnitude but narrowly crossed the null after adjustment. These findings argue against a simple dose-response model in which progressively greater anatomical incompleteness necessarily produces progressively greater neurological severity.

The timing analyses addressed an important concern created by the seven-day eligibility window. More than three-quarters of participants had NIHSS assessed within 48 hours and 88.4% within 72 hours. Restricting the analysis to ≤48 hours and ≤24 hours did not attenuate the estimated association; the adjusted coefficients were 2.193 and 2.612 NIHSS points, respectively. These analyses improve temporal comparability but do not eliminate residual uncertainty from last-known-well estimates, wake-up strokes, referral delays, or clinical changes before assessment.

Anatomical variation in regional context

The observed CoW distribution is biologically plausible and consistent with the marked anatomical variability reported in South Asian and Nepalese populations. Nepalese angiographic studies have repeatedly shown that posterior circulation and PCom abnormalities are common [13-15]. Dhakal et al. reported an anatomical CoW variation in 54.7% of patients undergoing cerebral CTA, with PCom hypoplasia the most frequent variant [13]. Sharma et al. found variant morphology in 53.4% of a Nepalese MRA cohort and noted PCom abnormalities in approximately two-thirds of abnormal circles [14]. More recently, Thapa et al. again demonstrated a predominance of posterior-circulation variants on 3-T MRA [15].

Comparable patterns have been reported elsewhere in South Asia. In a cadaveric study of 225 Sri Lankan adults, De Silva et al. found a typical CoW in only 14.2%, with bilateral PCom hypoplasia the most frequent individual configuration [18]. In a 300-patient South Indian MRA study, Naveen et al. reported a fully complete CoW in 16.6%, and bilateral PCom hypoplasia or absence was the commonest posterior configuration [19]. Direct comparison of prevalence percentages across studies should remain cautious because vessel-caliber thresholds, imaging modalities, source populations, and definitions of complete and incomplete differ substantially.

CoW integrity and stroke severity

The present primary finding is directionally aligned with the South Asian study that informed sample-size planning. Chowdhury et al. reported higher NIHSS scores among patients with incomplete CoW compared to those with complete CoW, with mean scores of 9.95 ± 5.10 and 6.60 ± 4.97, respectively (p=0.037), and an unadjusted OR of 4.50 (95% CI 1.18-18.13; p=0.029) for their more-severe stroke category [17]. Their severity threshold was not identical to the present NIHSS score >15 endpoint, and their analysis did not include multivariable adjustment.

Zhou et al. reported a Hartkamp distribution very similar to the present cohort and found higher admission NIHSS in patients with incomplete CoW [8]. However, the broader literature is not uniform. A systematic review and meta-analysis by Oumer et al. found a non-significant pooled association between CoW variation and ischemic stroke [9]. Lin et al. reported worse discharge outcome with incomplete CoW variants [10], whereas treatment-specific cohorts have sometimes found little or no independent relationship between a simple complete/incomplete classification and post-treatment outcome [11,12]. Chuang et al. reported greater early NIHSS improvement and better functional outcome with a complete CoW after intravenous thrombolysis [20], while Westphal et al. found no significant association between CoW variant status and NIHSS score at admission or functional outcome in patients with MCA-M1 occlusion undergoing endovascular treatment [21]. Sirvinskas et al. similarly emphasized the joint importance of CoW configuration and thrombus location [22].

The absence of a Hartkamp gradient in the present data is important. If simple anatomical incompleteness acted only through a graded reduction in global collateral capacity, one might expect Type IV to show greater severity than Types II or III and Type I to show the least severity. That pattern was not seen. Likewise, the exploratory regional analysis did not show that anterior-component incompleteness was more strongly associated with NIHSS score than posterior-only incompleteness; the overall regional tests were non-significant. Therefore, the binary association may reflect heterogeneous anatomical configurations, territory-specific hemodynamic relevance, or residual confounding rather than a single graded collateral mechanism. Anatomical completeness is not equivalent to functional collateral capacity: the effect of a communicating segment depends on whether it can supply the threatened territory, the pressure gradient across it, the site and burden of occlusion, and the wider leptomeningeal collateral network [23-26].

Clinical magnitude and interpretation

The adjusted complete-versus-incomplete difference of approximately 1.9 NIHSS points should be interpreted as modest. An increase of ≥4 NIHSS points has been used as one definition of neurological deterioration after acute ischemic stroke, although alternative thresholds have also been proposed [27]. Such within-patient change thresholds should not be interpreted as an established minimal clinically important difference for a cross-sectional between-group comparison. The present CI extended from 0.164 to 3.590 points, so its lower bound is compatible with a clinically negligible difference and even the upper bound remains below four points. This calibration, together with the adjusted R² of 0.073, indicates that CoW completeness accounts for only a small part of the variability in admission neurological severity.

Atrial fibrillation and neurological severity

Atrial fibrillation/flutter showed the strongest and most consistent clinical association with NIHSS in this study. This finding is concordant with longstanding population-based evidence that strokes occurring in the presence of atrial fibrillation are, on average, more severe [28]. In the Japan Stroke Data Bank, Toyoda et al. reported a median NIHSS score of 9 in patients with atrial fibrillation and 3 in those without atrial fibrillation, with an adjusted 5.468-point higher NIHSS score in atrial fibrillation [29]. They also reported an adjusted OR of 3.799 for their severe-stroke definition, but their threshold was NIHSS ≥10, which is substantially lower than the NIHSS >15 endpoint used in the present study and therefore should not be treated as directly comparable. Pre-stroke anticoagulation may also modify this phenotype [30], although anticoagulation status and stroke mechanism were not comprehensively modeled in the present analysis.

Clinical implications

CTA in acute ischemic stroke provides more than identification of a major arterial occlusion; it also depicts the architecture of potential primary collateral pathways. The present findings suggest that CoW completeness may add contextual information when interpreting initial stroke severity. However, the modest primary effect, limited model explanatory power, absent Hartkamp gradient, and non-significant regional tests argue strongly against using CoW completeness in isolation. Clinically meaningful assessment should integrate occlusion site and burden, leptomeningeal collaterals, perfusion status where available, infarct extent, baseline NIHSS, stroke mechanism, reperfusion treatment, and systemic factors such as atrial fibrillation. The current data do not support using CoW completeness alone as an individual prognostic classifier or treatment-selection criterion.

Strengths and limitations

This study has several strengths. It employed a prespecified primary continuous outcome, a standardized CTA acquisition protocol, a clearly defined Hartkamp classification framework, and complementary nonparametric and multivariable statistical approaches. The study flow was explicitly documented, with 462 patients assessed for eligibility, 190 enrolled, and all enrolled participants included in the final analysis. Additional analyses included influence diagnostics, restriction to anterior-circulation stroke, timing-restricted models, a parsimonious logistic regression model, and exploratory assessment of regional CoW morphology. Furthermore, participants with complete and incomplete CoW configurations were broadly comparable with respect to measured demographic characteristics and major vascular risk factors, reducing the likelihood that the observed association was attributable to substantial baseline imbalance in these measured variables.

Several limitations should be considered. First, this was a single-center, tertiary-care study restricted to patients aged >50 years who underwent CTA, which limits generalizability to younger patients, individuals with acute ischemic stroke who do not undergo CTA, and populations managed in other healthcare settings. The eligibility process also introduced potential selection effects because patients without adequate CTA imaging and those who had received reperfusion therapy or another intervention before baseline neurological assessment were excluded. These criteria may have preferentially excluded some rapidly presenting patients or those with particularly severe strokes.

Second, the observational design permits assessment of association but does not establish causality. Several potentially important stroke-related variables were not available for comprehensive adjustment, including precise occlusion location and clot burden, Alberta Stroke Program Early CT Score (ASPECTS) or infarct volume, standardized leptomeningeal collateral scores, stroke mechanism, pre-stroke anticoagulant use, and longitudinal functional outcomes. Residual confounding therefore remains possible, although adjustment for the measured covariates did not materially alter the estimated association between CoW completeness and NIHSS.

Third, onset-to-assessment timing was based on symptom onset or last-known-well time and was uncertain in some patients with wake-up stroke. In addition, approximately one-third of participants were interfacility referrals, creating the possibility that neurological status evolved before assessment at the study center. Analyses restricted according to assessment timing yielded findings consistent with the primary analysis, but they cannot fully account for changes in stroke severity occurring before neurological evaluation.

Fourth, CoW classification was performed by a resident and confirmed by a supervising radiologist rather than through independent, blinded assessment by two separate readers. Independent reader-level classifications were not available, precluding formal assessment of interobserver agreement. Although the final CoW classification was performed without access to the numeric NIHSS score, complete blinding to all clinical information available during routine care could not be assured.

Fifth, although thin-section CTA with multiplanar, maximum-intensity projection, and volume-rendered reconstructions was used for CoW assessment, classification of very small arterial segments remains subject to technical limitations. In particular, the 0.8-mm threshold used to define hypoplasia lies within a range in which measurements may be influenced by spatial resolution, partial-volume effects, contrast opacification, and reconstruction parameters. Furthermore, no standardized repeat-measurement protocol, intermeasurement reproducibility assessment, or phantom-based validation was performed. Therefore, some misclassification of vessels with calibers close to the 0.8-mm threshold cannot be excluded.

Sixth, the NIHSS is less sensitive to several neurological deficits arising from posterior-circulation ischemia. This is particularly relevant because abnormalities involving posterior CoW components accounted for a substantial proportion of incomplete configurations. Restriction of the analysis to anterior-circulation stroke reduced this concern, although the adjusted confidence interval in that analysis narrowly included the null value.

Seventh, the sample size calculation was based on the primary continuous NIHSS score comparison under an equal-allocation assumption, whereas the observed ratio of complete to incomplete CoW configurations was approximately 1:3. The secondary logistic analysis was not separately powered. Only 40 participants had an NIHSS score >15, corresponding to approximately eight events per predictor in the five-variable logistic regression model. Although the parsimonious model yielded a similar point estimate, the relatively wide confidence interval indicates limited precision and warrants cautious interpretation of the secondary dichotomous outcome.

Finally, no formal adjustment for multiple comparisons was applied. Analyses involving Hartkamp subgroups, rank-based comparisons, regional morphology, and other secondary or sensitivity analyses should therefore be regarded as exploratory and descriptive rather than as independent confirmatory findings.

## Conclusions

Incomplete CoW anatomy was common among patients aged >50 years with acute ischemic stroke undergoing CTA at a tertiary center in Nepal, with posterior-component abnormalities predominating. Incomplete CoW was associated with a modestly higher NIHSS score at admission after adjustment for measured covariates, but the primary model explained only a small proportion of NIHSS variability, and the lower confidence bound was compatible with a clinically negligible effect. The absence of a monotonic Hartkamp severity gradient, the non-significant regional morphology tests, and the borderline anterior-circulation sensitivity result indicate that binary anatomical completeness should not be equated with functional collateral capacity.

The secondary association with NIHSS score >15 was larger in relative terms but imprecise because of the limited number of events. Atrial fibrillation/flutter showed a stronger and more consistent association with admission severity. CoW morphology should therefore be interpreted within a broader vascular and clinical framework, not as a stand-alone prognostic or treatment-selection marker. Larger multicenter studies incorporating occlusion-site analysis, quantitative collateral and perfusion assessment, infarct volume, stroke mechanism, treatment, reader-reliability testing, and longitudinal functional outcomes are needed to determine which specific CoW configurations have clinically meaningful relevance.
